# Vitamin D Maintains Growth and Bone Mineral Density against a Background of Severe Vitamin A Deficiency and Moderate Toxicity in a Swine Model

**DOI:** 10.3390/nu16132037

**Published:** 2024-06-27

**Authors:** Cacious B. Phiri, Christopher R. Davis, Michael Grahn, Bryan M. Gannon, Brittney P. Kokinos, Thomas D. Crenshaw, Sherry A. Tanumihardjo

**Affiliations:** 1 Nutrition and Metabolism Graduate Program, Department of Nutritional Sciences, University of Wisconsin-Madison, Madison, WI 53706, USA; cphiri@wisc.edu (C.B.P.); bgannon@wisc.edu (B.M.G.); 2 Department of Animal and Dairy Sciences, University of Wisconsin-Madison, Madison, WI 53706, USA; kokinos@wisc.edu (B.P.K.); tdcrensh@wisc.edu (T.D.C.)

**Keywords:** kyphosis, liver, retinol, retinyl esters, toxicity

## Abstract

Excessive vitamin A (VA) negatively impacts bone. Interactions between VA and vitamin D (VD) in bone health are not well-understood. This study used a traditional two-by-two factorial design. Pigs were weaned and randomized to four treatments (*n* = 13/group): −A−D, −A+D, +A−D, and +A+D for 3 and 5 wk. Serum, liver, kidney, adrenal glands, spleen, and lung were analyzed by ultra-performance LC. Growth was evaluated by weight measured weekly and BMD by DXA. Weights were higher in −A+D (18.1 ± 1.0 kg) and +A+D (18.2 ± 2.3 kg) at 5 wk than in −A−D (15.5 ± 2.1 kg) and +A−D (15.8 ± 1.5 kg). Serum retinol concentrations were 0.25 ± 0.023, 0.22 ± 0.10, 0.77 ± 0.12, and 0.84 ± 0.28 µmol/L; and liver VA concentrations were 0.016 ± 0.015, 0.0065 ± 0.0035, 2.97 ± 0.43, 3.05 ± 0.68 µmol/g in −A−D, −A+D, +A−D, and +A+D, respectively. Serum 25(OH)D_3_ concentrations were 1.5 ± 1.11, 1.8 ± 0.43, 27.7 ± 8.91, and 23.9 ± 6.67 ng/mL in −A−D, +A−D, −A+D, +A+D, respectively, indicating a deficiency in −D and adequacy in +D. BMD was highest in +D (*p* < 0.001). VA and the interaction had no effect on BMD. Dietary VD influenced weight gain, BMD, and health despite VA status.

## 1. Introduction

Osteoporosis is a major public health problem characterized by compromised bone strength that predisposes individuals to an increased risk of fracture [1,2]. The prevalence of osteoporosis in the US was 10.3% among adults aged > 50 y in 2014 [3]. Important nutrients in bone development are calcium, phosphorous, vitamin D (VD) [4,5,6], and vitamin A (VA) [7]. Vitamins A and D play important roles in calcium homeostatic pathways involving osteoclast and osteoblast activities [8,9]. Therefore, maintaining an adequate supply of these micronutrients is essential for bone development. Vitamin D is important for bone formation and increases bone mineralization [4]. Mechanistically, 1,25(OH)_2_D_3_ (calcitriol), the active form of VD, increases intestinal dietary calcium absorption and mobilizes calcium from the bone to maintain plasma homeostasis [4]. Vitamin A status is known to be important in bone formation, and both hypo- and hypervitaminosis A have profound adverse effects [10]. Excessive VA intake reduced bone mineralization in rats [11] and humans [1,2,12]. Pigs with hypovitaminosis A showed paralysis and lack of coordination in pelvic limbs [13], lethargy, and seizures [14]. These findings demonstrate that an appropriate VA intake is needed for the maintenance of bone mineralization and bone health.

Vitamin A deficiency is a major public health concern affecting approximately 190 million preschool children and 19 million pregnant women [15]. Vitamin A supplementation is recommended in children to prevent mortality and xerophthalmia [16]. Interventions such as food fortification, biofortification, supplementation, and dietary diversification are implemented to ensure adequate intake [17]. These interventions often target the same vulnerable individuals, suggesting the possibility of high intake leading to hypervitaminosis A, which was reported in the USA, Zambia, and South Africa [18]. In low-income countries such as Malawi, VA deficiency declined from 59.2% in 2001 to 3.6% in 2015 [19] with evidence of excessive VA status among under-five-year-old and school-age children due to overlapping interventions (i.e., supplementation and fortification) [20]. Moreover, VD deficiency is documented in many countries. Vitamin D deficiency was 25.6% among healthy adults in Germany [21] and 36% in the USA [5], implying that it is a global problem.

Cross-sectional and longitudinal studies of VA and bone health show conflicting results. Most studies demonstrate that excessive VA intake reduces bone health and increases the risk of bone fracture [1,2]. The interaction of VA and VD in bone health has not been thoroughly elucidated, despite the known individual effects. This study investigated the interrelationships of excessive or deficient intakes of VA and VD on bone formation and growth using a pig model. Pigs have biological similarities to humans in anatomical size and structure, physiology, and immunology, and are an excellent model to understand certain diseases [22]. This study is unique because both excessive and deficient intakes of VA and VD were used, with a traditional two-by-two factorial design. Little is known about the VA and VD requirements of swine used as a biomedical model. Our hypothesis was that excessive VA in combination with excessive VD would lead to bone abnormalities. We further hypothesized that excessive VA would rescue the negative growth effects of VD deficiency.

## 2. Materials and Methods

Procedures with swine were approved by the College of Agricultural and Life Sciences Animal Care and Use Committee, University of Wisconsin–Madison (Protocol A005679-R01; initial approval 29 November 2019 for three years). Young pigs (*n* = 52) were weaned at 17 d from crossbred females (Landrace X Large White) mated with a Duroc boar were selected at the Swine Research and Teaching Center in Arlington, Wisconsin. This facility is enclosed, and the pigs had no exposure to ultraviolet light B in the range of 280–315 nm from natural or supplemental lighting [23], thereby preventing VD synthesis from light. Following typical herd management protocols, males were castrated shortly after birth. Weights were recorded at birth, a day before weaning, and once every week until the end of the intervention. Pigs were allocated to pens by treatment group and fed ad libitum until the night before termination. The study followed a two-by-two factorial design with low/high A and low/high D, resulting in four treatment groups (−A−D, −A+D, +A−D, +A+D). Pigs were randomized to treatment pens (*n* = 13/treatment, three pens/treatment) with blocking according to litter, sex, and weight. Pigs were fed ad libitum until being fasted ≥ 18 h before termination.

Two types of feeds were prepared for Phases 1 and 2 (Table 1). The pigs were fed nursery feed for 2 wk and thereafter grower feed for 3 wk for a total of 5 wk. Pen feed consumption was measured weekly. The source of VA was Microvit A (Adisseo North America, Inc.; Alpharetta, GA, USA), and that of VD was Rovimix D (DSM North America, Inc., Parsippany, NJ, USA). Microvit provides 1,000,000 IU per g VA as retinyl acetate, while Rovimix provides 500,000 IU D_3_ per g. Aliquots of the concentrated sources were premixed with other vitamins and trace minerals, before the addition of the premix to the major feed ingredients. Amounts of 0.01 g and 15.5 g of VA fortificant were added to 100 kg of complete feed to provide either 100 IU or 155,000 IU/kg feed for the low- and high-VA treatment groups, respectively. The low-VA value was meant to keep the pigs alive but not result in significant liver storage. The high-VA value was estimated to result in a liver concentration that was close to 3 μmol/g, a value associated with abnormal liver histology in humans [18]. Amounts of 0.008 g and 0.8 g of the Rovimix of vitamin D_3_ fortificant were added to 100 kg complete feed to provide 40 IU and 4000 IU/kg in the low- and high-VD treatment groups, respectively. In wk 4, the low-VD feed was increased to 0.028 g to provide 140 IU D_3_/kg feed, because some pigs in this group became lethargic and kyphotic. Very little is known about the VD requirements of swine that do not have access to sunlight.

Baseline pigs (*n* = 3/treatment) were killed at 3 wk (d 21 after feeding the dietary treatments) using approved captive bolt methods. The remaining 10 pigs/treatment were killed at 5 wk (d 35), which was randomized by pig among groups to balance termination times across treatment groups. Venous blood (~10 mL) was collected from the neck during restraint. The blood was centrifuged at 1000× *g* for 20 min to separate serum and was kept on dry ice before long-term storage at −80 °C at the University of Wisconsin–Madison. Tissues (i.e., liver, kidney, spleen, lung, and adrenal glands) were collected and kept on dry ice until storage at −80 °C. Right femurs were excised and stored at −20 °C.

Analyses of serum and tissues were performed using ultra-performance LC (UPLC) under yellow light to avoid vitamin losses [24]. The analysis included both retinol and retinyl esters, which were summed to obtain the total VA in the tissues. The serum retinol analysis was modified from Riabroy et al. [25], using 750 µL serum denatured with 750 µL ethanol (0.1% butylated hydroxytoluene as antioxidant). Then, 40 µL C23-β-*apo*-carotenol was added as an internal standard, and the sample was extracted twice with 2 mL mixed hexanes. The dried samples were reconstituted in 40 µL 75:25 methanol–dichloroethane.

The liver and extrahepatic tissues were analyzed, with modifications to sample weight and solvent volumes for extraction [25]. The sample weights were 1.0 and 1.5 g for the liver and kidney, respectively, and 3 g for spleen and lung. The liver was sampled from all four lobes. One adrenal gland was extracted with varying weights. A total of 50 mL methylene dichloride was used to extract the liver and kidney, while 25 mL was used for lungs, spleen, and adrenal glands. Five mL from −A and 0.5 mL for +A livers and 10 mL from lung, kidney, spleen, and adrenal gland extracts were dried under nitrogen. The difference in sample weight and volume extracted was due to differences in VA content among the tissues [25].

The femur was used for the analysis of bone mineral density (BMD) and bone mineral content (BMC). A dual-energy X-ray absorptiometry (DXA, GE Lunar Prodigy, Waukesha, WI, USA) instrument was used to scan (in small animal scan mode) the excised femurs as described previously [23,26]. The scans were analyzed using enCORE software (software version 12.20) to predict the bone area (cm^2^), BMC (g bone ash of the entire femur), and BMD (g/cm^2^, BMC/area). The BMD measure provides for correction in animals of different sizes, but the true volume of bone was not measured.

Data were analyzed using R (R Core Team, 2021, Vienna, Austria). Descriptive statistics are presented as means ± SDs. One pig died (+A−D) on d 20 and is not included in any statistical analyses. We modeled growth against treatment and controlled feed intake. After noting that treatment had no effect, we modeled feed intake against treatment to identify if the effect on weight gain was due to feed intake or treatment. A generalized linear regression was used to identify important variables that determine growth and BMD. The model was built using stepwise selection criteria, including VA treatment, VD treatment, serum retinol, serum VD, liver VA, and extrahepatic VA. The main effects of VA, VD, sex, and VA*VD interaction were tested for weight gain and BMD. A time factor was used to compare differences in biomarkers and variables between pigs at 3 and 5 wk. Fisher’s Least Significant Difference was used to determine differences in feed intake, serum retinol, serum VD, liver VA, extrahepatic VA, BMD, BMC, and weight gain among the treatment groups. Results were considered significant at *p* ≤ 0.05.

## 3. Results

### 3.1. Feed Intake, Growth, and Kyphosis

**Figure 1:**
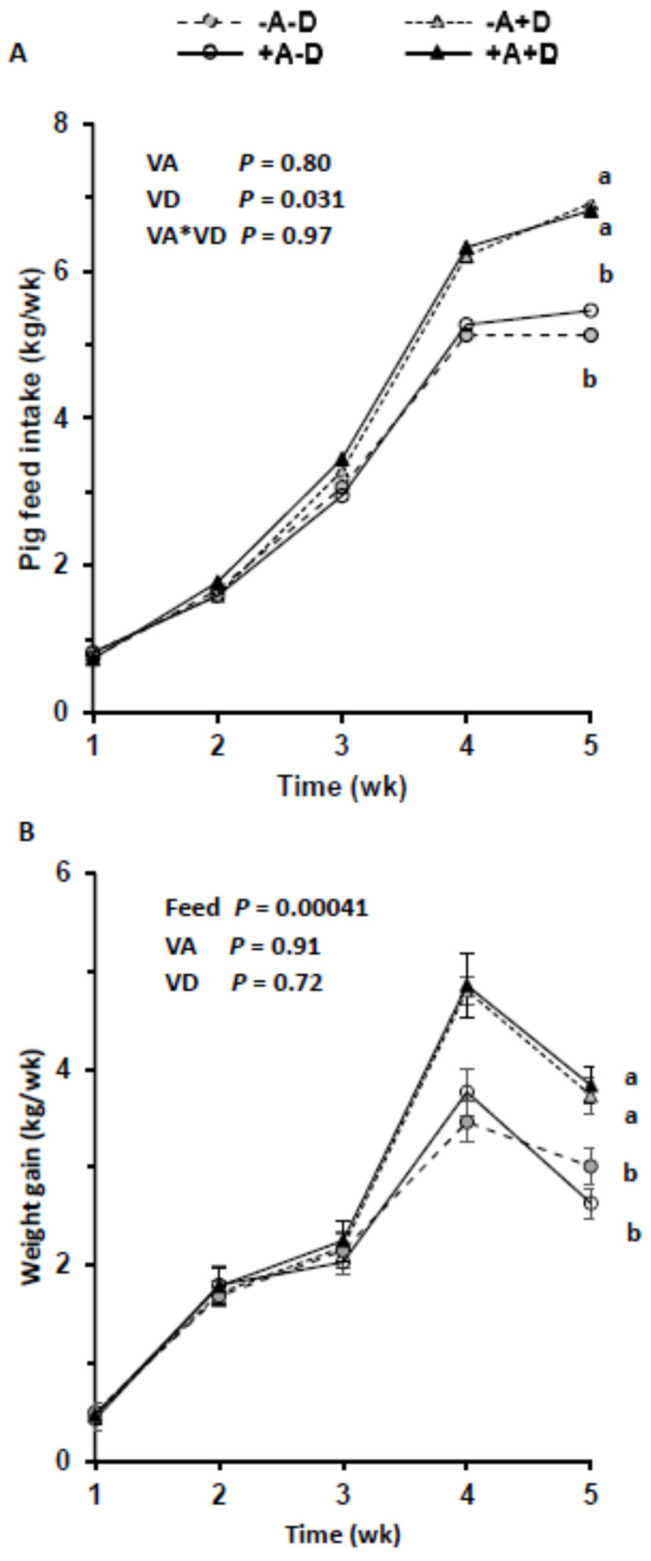
Mean feed intake per pig (*n* = 13/group) across treatment groups (**A**) and weekly weight gain across treatments (**B**). Each treatment included three pens. Pigs were allocated to a feed containing either −A−D, −A+D, +A−D, or +A+D. Groups labeled with different letters were significantly different, with a > b (*p* < 0.05). Significant differences were observed from wk 4 between +D and −D (*p* = 0.047; *p* = 0.0012, respectively). Vitamin A had no effect on feed intake (*p* = 1.0). Values in B are means ± SDs. −A−D, low A, low D; −A+D, low A, high D; +A−D, high A, low D; +A+D, high A, high D.

**Figure A1:**
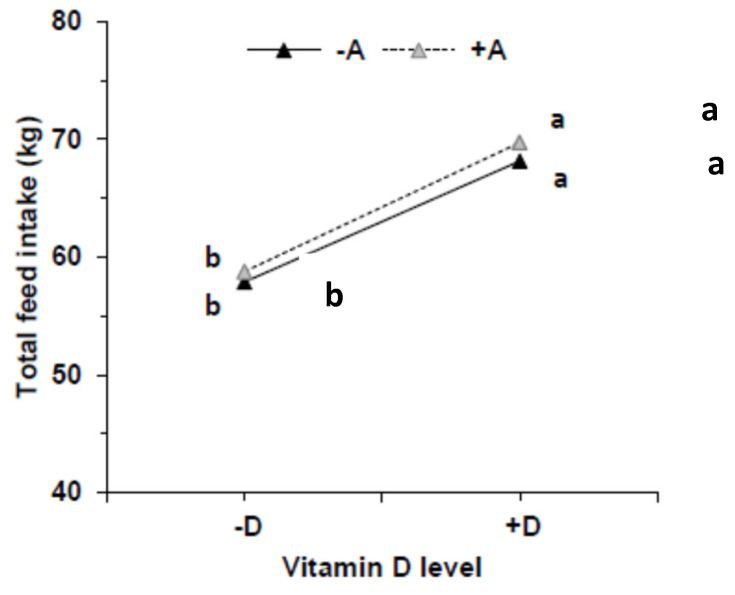
Feed intake from pigs that were fed feeds containing either −A−D, −A+D, +A−D, or +A+D (*n* = 10/group). Feed intake was significantly higher in groups that were fed high vitamin D (+D) (*p* = 0.0071). Vitamin A had no effect and the interaction with vitamin D did not affect feed intake (*p* = 0.65, *p* = 0.99, respectively). Groups labeled with different letters are significantly different with a > b. −A−D, low vitamin A, low vitamin D; −A+D, low vitamin A, high vitamin D; +A−D, high vitamin A, low vitamin D; +A+D, high vitamin A, high vitamin D.

By design, the dietary concentration of VA was below the requirement of 2200 IU/kg feed [27] in the −A groups and above in +A throughout the 5 wk. Similarly, the mean VD intake was below the requirement of 220 IU/kg feed in the −D groups and above requirements in the +D groups. Feed intake increased over time, as expected, with growth requirements and was highest in the +A+D group (Figure 1A), with the exception of wk 1. Feed intake was significantly associated with the level of VD in the feed (*p* = 0.031), but VA and the interaction of VA and VD did not influence feed intake (*p* = 0.80, *p* = 0.97, respectively; Appendix A, Figure A1). The interaction of VD with time was significant (*p* < 0.0001).

Weekly weight gain did not differ across the treatment groups until wk 4 (Figure 1B). Vitamin D influenced the mean weight gain (*p* = 0.011), but VA at the levels fed did not (*p* = 0.64). However, feed intake impacted the mean weight gain (*p* = 0.00041) when treatment was included in the model. In this model, VA, VD, and their interaction did not impact the mean weight gain (*p* = 0.91, *p* = 0.72, *p* = 0.81, respectively). Similarly, sex did not impact total weight gain (*p* = 0.65), but its interaction with VD was significant (*p* = 0.0068).

One pig in the −A−D and three pigs in +A−D, were kyphotic and lethargic by d 21. These pigs were weak and had challenges standing, usually taking a dog-sitting position. Previous studies showed that kyphosis was associated with VD deficiency [23,28].

### 3.2. Liver Vitamin A

**Figure 2:**
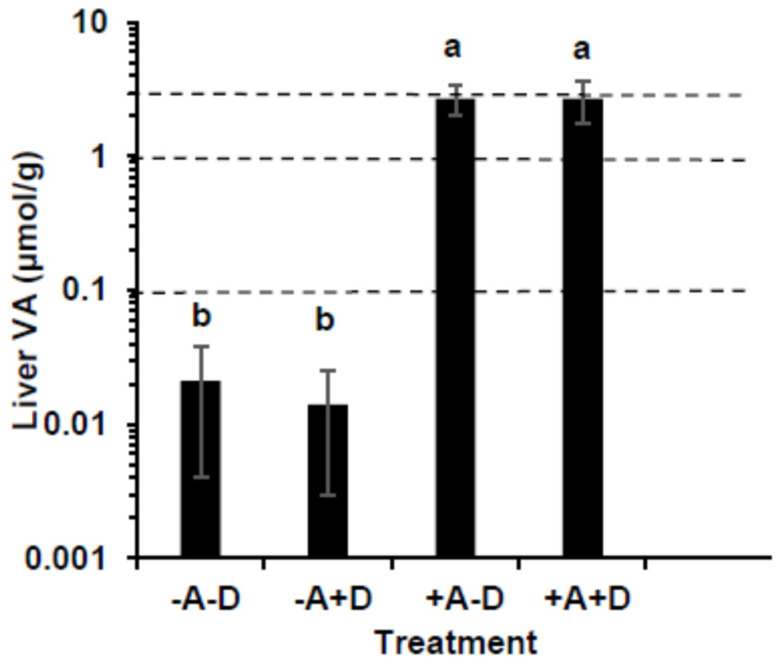
Comparison of liver vitamin A (VA) concentration in swine (*n* = 13/group, except group +A−D, where *n* = 12) that consumed treatment feeds consisting of −A−D, −A+D, +A−D, and +A+D. The dotted lines represent cut-off points of VA status; 0.10 indicates VA deficiency, 0.1–1.0 µmol/g represents normal, ≥3.0 µmol/g represents toxicity. All values are means ± SDs, and groups labeled with different letters are significantly different with a > b (*p* < 0.05). The +A groups were hypervitaminotic, with many having high circulating retinyl esters, a biomarker of toxicity; the −A groups were deficient. −A−D, low A, low D; −A+D, low A, high D; +A−D, high A, low D; +A+D, high A, high D.

Liver VA concentrations were significantly higher in +A groups than −A (Figure 2, *p* < 0.001). The mean liver total VA was below 0.10 µmol/g in all pigs in both −A groups, indicating VA deficiency. The mean liver VA concentrations in the +A groups were 2.71 ± 0.71 and 2.68 ± 0.92 µmol/g, indicating hypervitaminosis A. The proportion as retinyl esters was >90% in the liver. All pigs except one in each of the +A groups had hypervitaminosis A defined as ≥1 μmol/g liver. The livers were screened for retinyl acetate, the dietary form, and only traces were detected. Total VA in the liver was highest in +A+D and lowest in −A+D. Vitamin A impacted the total VA (*p* < 0.0001), but VD and their interaction did not.

### 3.3. Serum Retinol, Retinyl Esters, and Vitamin D

**Figure 3:**
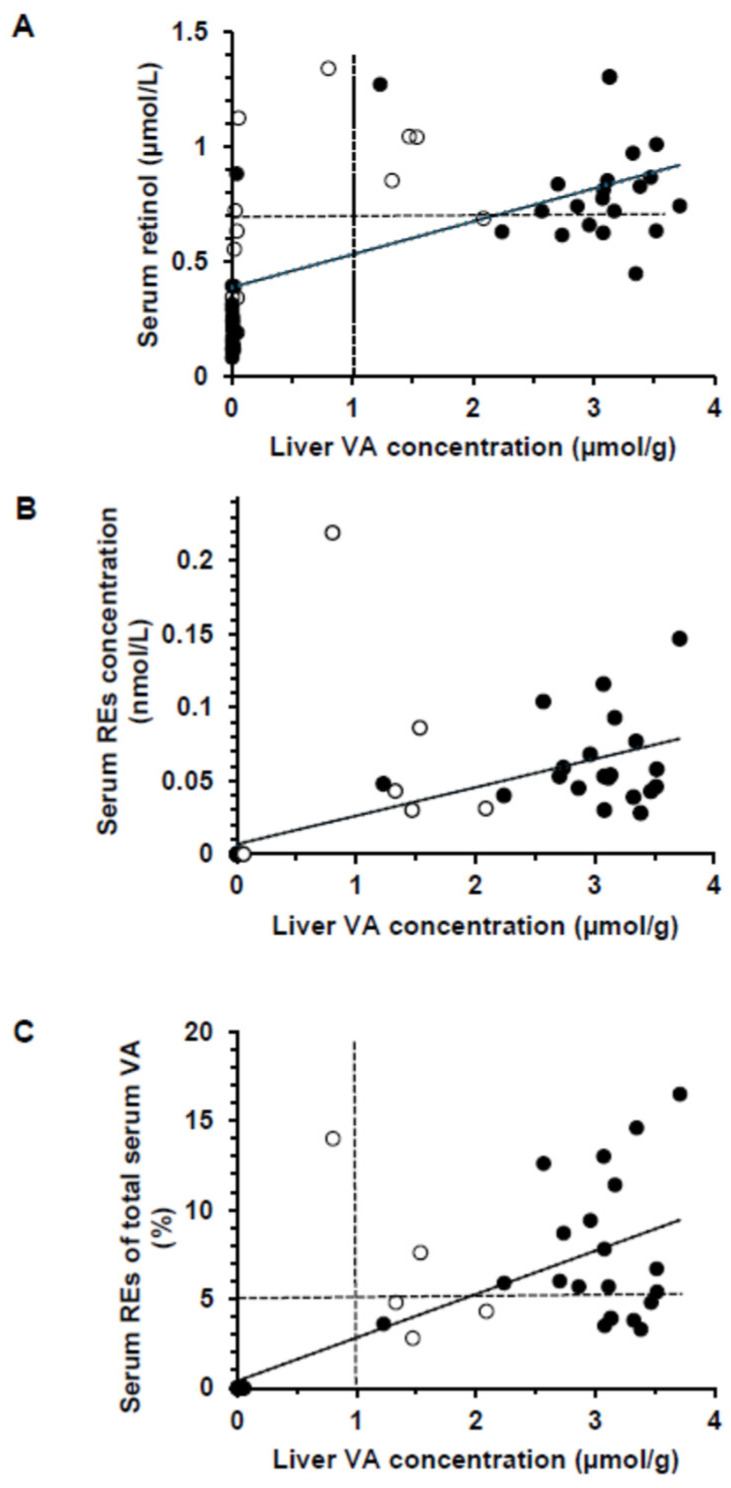
Scatterplots showing the relationship between the vitamin A (VA) concentration in the liver in all piglets [*n* = 51, which includes wk 3 (empty circles)] and those killed at wk 5 (*n* = 40, black circles). (**A**) Serum retinol compared with liver VA concentration for all pigs (*r* = 0.594) and those from wk 5 only (*r* = 0.779, *p* < 0.0001). The dashed line represents the serum retinol cut-off point for VA deficiency (0.70 µmol/L). (**B**) Serum retinyl ester (RE) concentration plotted against liver VA concentrations for all pigs (*r* = 0.633) and those from wk 5 only (*r* = 0.829, *p* < 0.0001). (**C**) Serum VA as percentage RE of total serum VA compared with liver retinol concentration (*r* = 0.766) and those from wk 5 only (*r* = 0.817, *p* < 0.0001). The dashed lines are the current cut-off points for hypervitaminosis A (>1 µmol/g liver) and the 5% used for children for the percentage of total serum retinol as retinyl esters.

The mean serum retinol concentration was significantly higher in +A than in −A, regardless of VD status (*p* < 0.0001, Table 2). By design, pigs were severely VA deficient in −A groups, with a mean serum retinol ≤ 0.70 µmol/L regardless of time (Figure 3A). Nonetheless 7 pigs in the +A groups had serum retinol concentrations ≤ 0.70 µmol/L, defined as false positives for indicating VA deficiency relative to liver VA. Therefore, serum retinol concentrations indicated 88.5% and 28% VA deficiency in the −A and +A groups, while liver VA concentrations indicated 100% and 0% deficiency, respectively. In the +A groups, the concentration of serum retinol was higher in pigs killed at 5 wk than 3 wk (*p* < 0.0001). No serum retinyl esters were observed in −A at the time of killing (Figure 3B). Feed was pulled at least 18 h prior to blood collection, indicating that the retinyl esters were not from recent dietary intake in the +A groups. Retinyl acetate, which was the form of VA in the feed, was only detected in the serum of the pig that died on d 20 and was not fasted. A total of 75% and 53.8% of the pigs had circulating serum retinyl esters above 5% of the total VA in +A−D and +A+D, respectively, which is a biomarker of VA toxicity [18,20] (Figure 3C).

The reference serum VD concentration in growing pigs aged 2 wk to 8 mo is 18–30 ng 25(OH)D/mL [6]. All pigs in the −D groups, two in −A+D, and three in +A+D were deficient by this parameter. The mean serum VD concentrations were deficient in the −D groups and adequate in the +D groups (Table 2). Serum 25(OH)D was significantly affected by time (*p* < 0.0001).

### 3.4. Extrahepatic Vitamins A and D

**Figure 4:**
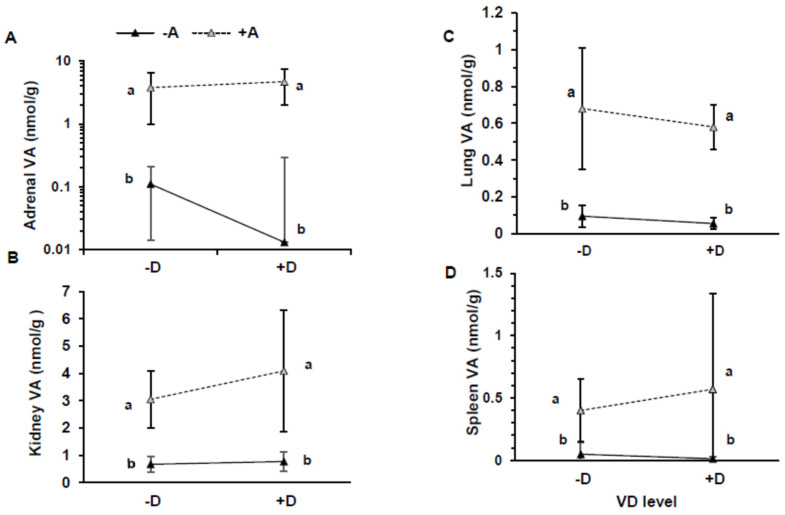
Vitamin A (VA) concentration in adrenals (**A**), kidneys (**B**), lung (**C**), and spleen (**D**) in pigs that consumed treatment feeds consisting of −A−D, −A+D, +A−D, +A+D. All values are means ± SDs, and groups labeled with different letters are significantly different, with a > b (*p* < 0.05). Concentration of VA was higher in +A treatments than −A treatments. *n* = 13/group in all groups, except *n* = 12 in +A−D, and *n* = 12 for kidneys in −A+D. −A−D, low A, low D; −A+D, low A, high D; +A−D, high A, low D; +A+D, high A, high D.

The extrahepatic tissues showed higher VA concentrations in the +A groups than the −A groups [Figure 4A–D; (all *p* ≤ 0.0001, except for the lungs *p* = 0.013)]. The lowest concentration was in the −A−D group, and the highest was in the +A+D group. The highest concentration was found in the adrenals, followed by the kidneys, lungs, and spleen. Total organ VA was highest in kidney, followed by lung and spleen (Table 3), and the contributing factors were tissue concentration and size. Vitamin D was not quantifiable by UPLC in the tissues analyzed.

### 3.5. Bone Mineral Density and Content

**Figure 5:**
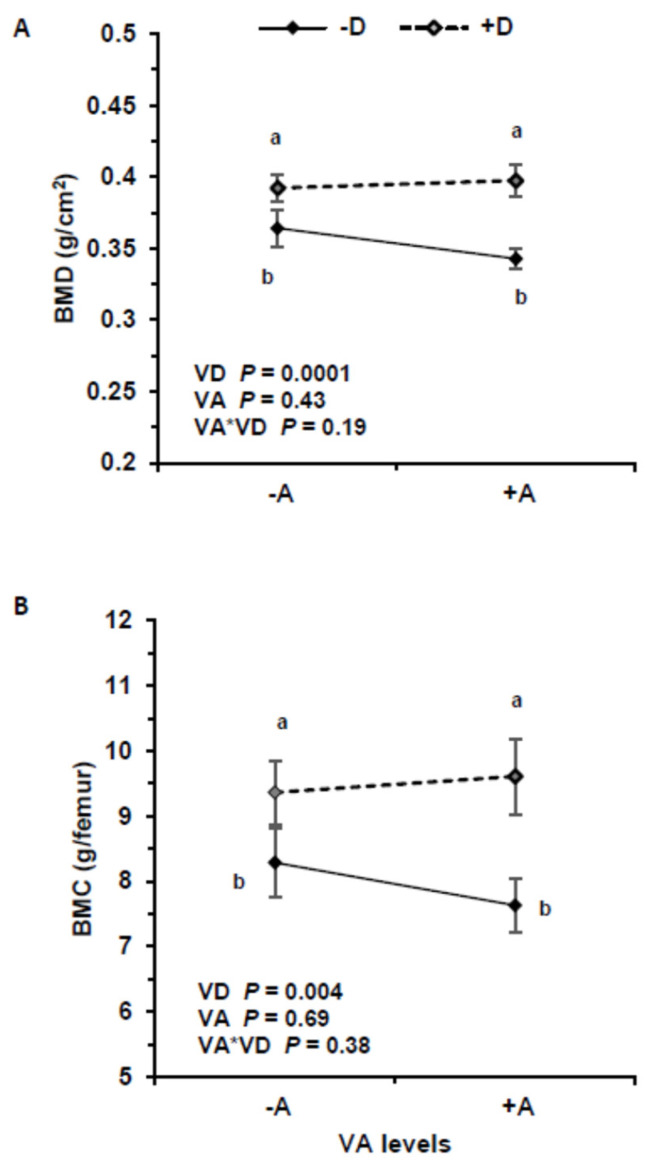
Bone mineral density (BMD) (**A**) and bone mineral content (BMC) (**B**) in pigs that consumed treatment feeds consisting of −A−D, −A+D, +A−D, +A+D (*n* = 13/group in all groups, except *n* = 12 in +A−D). All values are means ± SDs, and groups labeled with different letters are significantly different with a > b (*p* < 0.05). BMD was highest in +D treatments. Vitamin A had no effect on BMD and BMC. −A−D, low A, low D; −A+D, low A, high D; +A−D, high A, low D; +A+D, high A, high D.

The highest BMD was recorded in the +A+D group (0.398 ± 0.039 g/cm^2^) and the lowest in +A−D group (0.341 ± 0.027 g/cm^2^) (Figure 5). The BMD was different between groups +A−D and +A+D (*p* = 0.0018) and +A−D and −A+D (*p* = 0.0053). Increasing VD intake was positively associated with BMD (*p* < 0.00022), and serum 25(OH)D_3_ was positively associated with BMD (*p* = 0.00014), but VA and the interaction had no effect on BMD (*p* = 0.44, *p* = 0.18, respectively). Serum retinol and liver VA had no association with BMD (*p* = 0.99, *p* = 0.85, respectively). Vitamin D affected BMD with similar responses at 3 and 5 wk. Vitamin A concentration in kidneys, adrenal, and spleen did not influence BMD (*p* = 0.91; *p* = 0.28; *p* = 0.45, respectively) but lung VA was positively associated with BMD (*p* = 0.01).

Similarly, VD intake significantly impacted BMC (*p* = 0.0043), but VA and the interaction were not significant (*p* = 0.69, *p* = 0.38, respectively). Serum 25(OH)D_3_ significantly impacted BMC (*p* = 0.00038), but serum retinol and the interaction were not significant (*p* = 0.079, *p* = 0.34, respectively).

## 4. Discussion

To our knowledge, this is the first study to evaluate the interaction of excessive and deficient intakes of VA and VD on bone health in a pig model. Growth and femur BMD were measured, along with the nutritional biomarkers serum retinol, retinyl esters, and 25(OH)D_3_ concentrations. Pigs are born with low serum VD concentrations and rely on fetal stores. A former study fed gestating sows 200 or 2000 IU VD/kg feed and concluded that the higher amount is needed to increase fetal stores and support bone strength [29]. In the current study, the pigs fed +D had the highest weight gain, demonstrating that VD is essential in bone mineralization and growth. The mean BMD (0.394 g/cm^2^) was lower in the current study than a previous study with adequate dietary VD (0.558 g/cm^2^) [23]. The pigs in that study were weaned 4 days later than the current study. A former study in rats demonstrated that all-*trans* retinoic acid’s bone resorptive activity was independent of orally administered cholecalciferol [30]. Nonetheless, human studies of excessive VA and hip fracture yielded inconsistent results, with some finding an association [31,32], while others did not [33,34]. In these studies [31,32,33,34], the indicator for VA was dietary intake. In humans, supplemental 1,25-dihydroxyvitamin D_3_ increased serum calcium, while the addition of retinyl palmitate reduced serum calcium when taken alone or in combination with vitamin D_3_ [35]. Therefore, VD deficiency may mask the effects of a high VA intake among individuals with deficient VD status.

Weight gain and feed intake were significantly higher in the +D treatment groups than the −D groups. The mechanisms through which VD promotes growth are not clear [36]. However, VD interacts with growth hormone [37] and is involved in calcium homeostasis [4]. Vitamin A did not affect weight gain at the deficient and high levels in this study. Other studies in rats fed 12.5 times the VA adequacy [38] and mice fed at the projected human tolerable upper limit or three times that value [39] did not find negative impacts on body weight when compared with controls. A linear association was found between VA intake and the decreased BMD of cortical bone and femurs in mice [39]. Excess VA reduced the periosteal and endocortical circumference of the bone, with the severity of the condition increasing with time [39]. Excessive VA caused poor bone growth in rats [11] due to increased bone resorption and decreased bone formation. Nonetheless, it is well-known that VA is essential for optimal growth.

Pigs in the −D groups developed lethargy and kyphosis. Lethargy is a health indicator of inadequate energy intake or weak bones [40]. Low serum VD results in low serum calcium and phosphate, promoting demineralization, leading to weakness [40]. Calcium stimulates oxidative phosphorylation, leading to energy production; therefore, VD deficiency impairs energy production from oxidative phosphorylation [41]. Moreover, VD improves insulin secretion through the regulation of undercarboxylated osteocalcin [42], allowing more glucose into cells and energy production from carbohydrate metabolism. In previous studies, VD deficiency caused by the maternal diet was associated with kyphosis, while inadequate VD in neonatal diets further increased the risk of spinal deformity in swine [28]. In a case report, severe VD deficiency caused kyphosis in two women [43]. Studies are needed to understand the mechanisms of VD status and kyphosis in mammals.

Although VD showed a significant positive relationship with BMD, the excessive intake of VA was not associated with BMD. Both liver and serum retinol had no association with BMD, despite near-toxic liver levels and circulating retinyl esters. Vitamin D supports BMD through the maintenance of calcium homeostasis [4]. Low blood calcium signals the parathyroid gland to release parathyroid hormone, which has three metabolic functions. First, parathyroid hormone increases the reabsorption of calcium ions, thereby increasing blood calcium. Second, it stimulates the conversion of 25(OH)D_3_ into its active form, 1,25(OH)_2_D_3_ (calcitriol) through the activation of CYP27B1 (cytochrome P450 family 27 subfamily B member 1) [44], which increases intestinal calcium absorption. Third, parathyroid hormone and calcitriol act at the bone level to induce calcium mobilization through a cascade of cellular responses. Furthermore, calcitriol regulates the differentiation, proliferation, mineralization, bone resorption, and maturation of osteoblasts and osteoclasts [45], suggesting that VD exerts both anabolic and catabolic functions.

Excessive VA is thought to impair VD function by competing for the shared heterodimer retinoid X receptor; thus, VD would be unable to mobilize calcium, leading to bone demineralization. Although the findings from this study do not show that excessive VA intake impairs bone development, cross-sectional and nested case–control studies showed an inverse relationship in women [1,2], but VA status was not measured in these studies. Clinical signs such as bone pain, refusal to walk, diffuse tender bony swelling of the forelimbs, skin changes, and abdominal pain were observed in a 4-year-old boy after the excessive intake of preformed VA [12]. Restricting preformed VA intake for a short period of time increased a biomarker of bone formation, i.e., procollagen type I *N-*propeptide, in Zambian children [46].

Both VA and VD actions clearly affect clinical symptoms of skeletal health, yet specific actions at the cellular level are not clearly established. Various local signals stimulate or inhibit bone formation and resorption. Among these are osteoprotegerin (OPG), RANKL, osteopontin, and transforming growth factor-β1. The established role of VA in bone physiology involves retinoic acid binding to retinoic acid receptors, which heterodimerize with retinoid X receptors to induce transcription [8]. Bone continuously remodels, which is normally coupled by signals to control osteoblast and osteoclast cell activity. High all-*trans*-retinoic acid downregulates OPG, while RANKL is upregulated in the osteoblastic genes, resulting in increased bone resorption [47]. While OPG was similar in osteoporotic patients compared with controls, serum VD concentrations were lower, and RANKL was higher [48]. OPG is a decoy receptor of RANKL, preventing its association with RANK and suppressing osteoclast activity [49]. Transforming growth factor-β1 induces the proliferation, differentiation, and migration of bone mesenchymal stem cells, which produce osteoblast cells that stimulate bone mineral formation [50].

Furthermore, matrix metallopeptidases (MMPs) maintain bone health by altering the interactions of osteoclasts and the bone matrix, and by stimulating osteoblast differentiation, migration, spreading, and survival [51]. Low MMP results in a decrease in osteoblasts, ultimately resulting in a decrease in bone mineralization and increased osteolysis. The tissue inhibitor of metalloproteinases works to reverse MMPs by promoting bone resorption [51]. The signaling of bone morphogenic protein genes increases osteoblastic gene differentiation and induces the expression of osteocalcin, thereby stimulating bone formation [52]. However, high retinoic acid reduces osteocalcin expression, which inhibits mineralization [53]. Thus, dietary VA needs to be in balance for optimal bone health.

The negative health effects of VA deficiency are well-documented. By design, the −A groups in this study were severely VA deficient, as indicated by both serum retinol concentrations < 0.35 μmol/L and liver VA concentrations < 0.06 μmol/g. In children dying from multiple causes, 34.2% (*n* = 366) had an underlying VA deficiency, defined as a liver VA concentration < 0.10 μmol/g [54]. This was more common than hypervitaminosis A (≥1.0 μmol/g liver), which occurred in 8.7% of children in the same evaluation. Although serum retinol concentrations are a common indicator used in population evaluations, the WHO cutoff of 0.70 μmol/L [55] also classified the +A groups with VA deficiency. That is, 28% of the +A pigs had serum retinol ≤ 0.70 μmol/L, and 20% is the threshold to define a severe public health concern [55]. Combining biomarkers, such as serum retinol concentrations, with a modified relative dose–response test and/or fasting serum retinyl ester concentrations can better define the actual VA status, instead of relying on serum retinol concentrations alone, such as was done in a survey of Malawian children [20].

Using a traditional two-by-two factorial design, this study was limited because it was conducted over a short period of time (5 wk), and we did not have an adequate dietary intake control group that provided the recommended requirements for VA and VD [27]. Our objectives were to establish a range of VA and VD statuses that spanned deficiency through to excess. Few studies have evaluated the actual VA and VD requirements of swine used in biomedical research. In many cases, the swine model is more translatable to humans than other rodent models [22]; nonetheless, human studies are needed to help define the importance of dietary factors on bone health. The interaction of the whole diet with bone health is important for understanding the roles of essential vitamins [56].

Human studies that found that an excessive intake of preformed VA reduced BMD were conducted over a long period of time [32]. Findings from a clinical review showed that excessive VA intake was associated with low BMD in observational studies [57], but a safe level of intake could not be determined. Human studies evaluating the interaction of VA and VD on BMD should include long-term dietary intake and sensitive biomarkers of vitamin status. Future research to determine their interactions should include the effect on divalent metals, such as Mn and Zn, because these are cofactors for MMP, and key biomarkers of bone formation such as osteocalcin, alkaline phosphatase, and procollagen type I *N-*propeptide to establish their molecular mechanisms.

## 5. Conclusions

In conclusion, we did not see an effect of VA alone, or an interaction of VA and VD, on growth and bone outcomes at the dietary levels fed. This could be because the −A groups had a very low VA intake, while the +A groups were toxic, both of which have negative effects on bone formation.

## Figures and Tables

**Table 1:** Ingredients and nutrient composition (as-is basis) of Phase 1 and Phase 2 feeds ^1^ fed to pigs, with two levels of vitamin A and two levels of vitamin D.

Ingredients, g/kg	Phase 1 Nursery	Phase 2 Grower
Corn grain	394.8	625.4
Soybean meal, 48% crude protein	307.2	315.7
Oat groats	150.0	-
Whey	100.0	-
L-Lysine HCl	2.5	3.0
Methionine	1.25	1.0
Threonine	1.0	1.0
Monocalcium phosphate	4.50	10.6
Calcium carbonate	5.24	8.31
Corn oil	20.0	20.0
Sodium chloride	3.50	5.00
UW VTM-G without A and D_3_ ^2,3^	10.0	10.0
Total	1000.0	1000.0

^1^ Feeds were formulated to supply requirements (NRC, 2012) for 3 to 5 kg (Phase 1, Nursery) and 5 to 11 kg (Phase 2, Grower) pigs, with the exceptions of vitamins A and D. ^2^ UW VTM-G without vitamins A and D_3_ is a custom-mixed vitamin–trace mineral mix for growth, which provides the following nutrients per kg of the complete diet: vitamin A, 0 IU; vitamin D_3_, 0 IU; vitamin E, 14 IU; vitamin K, 0.75 IU; niacin, 22 mg; pantothenic acid, 12 mg; riboflavin, 8 mg; vitamin B_12_, 33 μg; Cu (from copper sulfate), 1.5 mg; I (from ethylene-diamine dihydroiodide), 0.3 mg; Fe (from ferrous sulfate), 38 mg; Se (from sodium selenite), 0.2 mg; and Zn (from zinc oxide), 90 mg. ^3^ Phase 1 and 2 feeds included Microvit retinyl acetate A (from Microvit A, 1,000,000 IU/g, Adisseo, Inc.) and vitamin D_3_ (from Rovimix D, 500,000 IU/g, DSM North America, Inc.) added to the premix to supply either low (100 IU/kg) or high (155,000 IU/kg) vitamin A treatments or low (40 IU/kg) or high (4000 IU/kg) vitamin D_3_ for the respective dietary treatments.

**Table 2:** Serum retinol, retinyl ester (RE), and 25(OH)D_3_ concentrations and RE as a percentage of total vitamin A in pigs on four dietary treatments of vitamins A (VA) and D (VD). [ND, not detected].

Biomarker	Treatment Duration (wk)	Treatment Group ^1^				*p*-Value
		−A−D	−A+D	+A−D ^2^	+A+D	
Serum retinol ^3^ (µmol/L)	3	0.67 ± 0.18	0.75 ± 0.37	0.64 ± 0.085	1.09 ± 0.43	VA < 0.0001 VD = 0.32 VA*VD = 0.68
	5	0.20 ± 0.087	0.22 ± 0.10	0.81 ± 0.15	0.84 ± 0.27	
Serum RE ^3^ (µmol/L)	3	ND	ND	0.065 ± 0.030	0.093 ± 0.11	VA < 0.0001 VD = 0.16 VA*VD = 0.17
	5	ND	ND	0.051 ± 0.021	0.073 ± 0.035	
RE % of total ^3^ serum VA	3	ND	ND	6.2 ± 2.0	7.0 ± 6.0	VA < 0.0001 VD = 0.15 VA*VD = 0.17
	5	ND	ND	6.4 ± 2.8	8.9 ± 4.8	
Serum 25(OH)D_3_ (ng/mL)	3	3.15 ± 4.97	19.8 ± 4.20	2.16 ± 0.95	16.1 ± 5.47	VA = 0.31 VD < 0.0001 VA*VD = 0.19
	5	1.5 ± 1.11	27.7 ± 8.91	1.73 ± 0.59	23.9 ± 6.67	

^1^ Samples were collected from 2–3 pigs at 3 wk, and 10 pigs at 5 wk. ^2^ One pig died on day 20 and its data are not included. Analysis was based on 2 pigs at 3 wk. ^3^ Time-influenced serum retinol, RE, and VD concentrations.

**Table 3:** Total vitamin A in the liver and extrahepatic tissues of pigs that were placed on four dietary treatments with low and high vitamins A and D.

Tissue ^1^	Treatment Duration (wk)	−A−D	−A+D	+A−D ^2^	+A+D	*p*
Liver (µmol)	3	9.76 ± 3.19	10.3 ± 6.61	331 ± 95.6	426 ± 310	VA < 0.0001 VD = 0.28 VA*VD = 0.26
	5	5.9 ± 5.83	7.17 ± 12.4	1170 ± 331	1394 ± 392	
Kidney (nmol)	3	35.7 ± 13.9	34.2 ± 1.51 ^2^	75.1 ± 25.3	101 ± 55.6	VA < 0.0001 VD = 0.03 VA*VD = 0.06
	5	51.0 ± 24.9	68.0 ± 35.7	267 ± 65.3	418 ± 188	
Lung (nmol)	3	7.07 ± 1.66	8.72 ± 5.24	36.5 ± 2.13	46.8 ± 27.2	VA < 0.0001 VD = 0.57 VA*VD = 0.91
	5	18.1 ± 12.2	9.54 ± 5.71	125 ± 61.2	123 ± 44.6	
Spleen (nmol)	3	1.79 ± 2.02	0.41 ± 0.53	3.43 ± 0.74	10.2 ± 7.06	VA < 0.0001 VD = 0.44 VA*VD = 0.73
	5	0.57 ± 0.63	0.29 ± 0.46	15.6 ± 11.4	12.1 ± 3.40	

^1^ Samples were collected from 3 pigs/treatment at 3 wk, and 10 pigs/treatment at 5 wk. ^2^ *n* = 2 at 3 wk.

## Data Availability

Data may be available by contacting the senior author (SAT) after completion of the thesis of CBP.
